# Spontaneous Mutations Decrease Sensitivity of Gene Expression to Random Environmental Variation in *Caenorhabditis elegans*

**DOI:** 10.1371/journal.pone.0008750

**Published:** 2010-01-18

**Authors:** Charles F. Baer, Dee R. Denver

**Affiliations:** 1 Department of Biology, University of Florida, Gainesville, Florida, United States of America; 2 University of Florida Genetics Institute, University of Florida, Gainesville, Florida, United States of America; 3 Department of Zoology, Oregon State University, Corvallis, Oregon, United States of America; 4 Center for Genome Research and Biocomputing, Oregon State University, Corvallis, Oregon, United States of America; The University of Chicago, United States of America

## Abstract

**Background:**

Biological phenotypes are described as “canalized” if they are robust to minor variation of environment and/or genetic background. The existence of a robust phenotype logically implies that some underlying mechanism must be variable, in the sense of “able to vary”, in order to compensate for variation in the environment and/or genetic effects. Several lines of evidence lead to the conclusion that deleterious mutations predictably render morphological, developmental, and life-history traits more sensitive to small random environmental perturbations - that is, mutations de-canalize the phenotype.

**Methodology/Principal Findings:**

Using conventional dye-swap microarray methodology, we compared transcript abundance in a sample of >7,000 genes between four mutation accumulation (MA) lines of the nematode *Caenorhabditis elegans* and the common (unmutated) ancestor. There was significantly less environmental variance in the MA lines than in the ancestor, both among replicates of the same gene and among genes.

**Conclusions/Significance:**

Deleterious mutations consistently decrease the within-line component of variance in transcript abundance, which is straightforwardly interpreted as reducing the sensitivity of gene expression to small random variation in the environment. This finding is consistent with the idea that underlying variability in gene expression might be mechanistically responsible for phenotypic robustness.

## Introduction

One of the hallmarks of animal development is its robustness to small environmental and genetic perturbations. This robustness is often called “canalization” [1], [2], [3], by which it is meant that the same phenotypic trait develops under disparate environmental conditions (environmental canalization) or in different genetic backgrounds (genetic canalization). A useful measure of environmental canalization is the reciprocal of the environmental component of phenotypic variance, 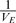 [3]; the smaller the environmental variance, the more canalized the trait is to environmental perturbations. In the lower extreme, if V_E_ = 0, the phenotype is the same no matter what the environmental conditions - it is completely canalized. An analogous measure of genetic canalization is the reciprocal of the mutational variance, 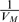; the smaller V_M_, the more robust the phenotype to mutational perturbation.

It has long been known that deleterious mutations increase V_E_ in certain circumstances, e.g., under inbreeding ([4], p. 289) and in genotypes with known mutations of large effect [5], [6]. We recently reported a study in which we quantified the change in V_E_ for fitness and body size with spontaneous mutation accumulation in three species of nematodes in the family rhabditidae [7]. Perhaps surprisingly, the magnitude of rate of change (increase) in V_E_ is approximately the same as the rate of change (decrease) of the traits themselves (fitness and body size). It has also been shown that spontaneous mutations increase the frequency of rare aberrant phenotypes in the development of the *C. elegans* vulva ([8]; C. Braendle, CFB, and M-A Felix, unpublished data). Two conclusions follow: there is abundant genetic variation for environmental variation [9], [10], and deleterious mutations consistently and predictably de-canalize morphological, developmental, and life-history traits.

Robustness of the phenotype to environmental perturbation logically implies that there must be variability, in the sense of “the ability to vary” at some underlying level [3], [11]. A convenient analogy is of a person driving a car on a winding, hilly road: to maintain a constant phenotype (e.g., driving down the road at constant speed), the system is necessarily variable at the underlying levels of steering, throttle, and brakes. An obvious possible mechanism by which living organisms may achieve this underlying control is via gene expression. Genes may be up or down-regulated in response to particular environmental perturbations, different combinations of genes may be expressed, etc.

As part of a comprehensive effort to characterize the cumulative effects of spontaneous mutations on environmental variance, we re-analyzed the data of Denver et al. [12], in which transcript abundance was measured in four lines of *C. elegans* in which mutations had accumulated for ∼280 generations and in the common ancestor of the mutation accumulation (MA) lines. Contrary to our *a priori* expectation, the accumulation of spontaneous mutations reduces the variability in gene expression, as measured by the environmental (residual) component of variance (V_E_) of transcript abundance.

## Materials and Methods

We refer the reader to the original publication [12] for details of the transcriptome analysis. Briefly, transcriptional variation was analyzed in four *C. elegans* MA lines and their common N2 progenitor using spotted PCR product arrays containing representative spots for >22,000 genes. Each genotype was represented by eight biological replicates in a dye-swap loop design. In the loop design each genotype was involved in two sets of competitive hybridization experiments (each against a different genotype), four biological replicates per pairwise genotype comparison. Each biological replicate represents the transcriptomes of many thousands of individual worms taken from age-synchronized populations at the young adult stage. For each biological replicate, worms were collected from 20 large-diameter NGM plates and subsequently pooled for total RNA extraction and poly(A) selection (single RNA extraction per replicate). Dye-labeled cDNA samples were hybridized to the array after which the arrays were scanned to obtain raw intensity values. Background correction and global LOESS normalization procedures were performed using R/MAANOVA; the normalized data used in subsequent analyses are the residuals from the first equation given in Box 3(a) in [13]. Normalized data are available online in Table S1.

### Data Analysis

The basic data structure is: 7056 unique genes are represented in each of four MA lines (MA24, MA41, MA83, MA99) and the common ancestral control (N2), each replicated eight times. For each gene we calculated the within-line (environmental) coefficient of variation (CV_E_, the standard deviation divided by the mean) of the eight normalized replicates in each line. The within-line component of variance includes variation among the many thousands of worms within a plate and variation among the 20 plates within a replicate. The within-line component of variance also includes a small amount of genetic variance contributed by segregating alleles. The expected time to fixation/loss of a new mutation with selective effect *s*<25% (4*N* *_e_s*<1) is two generations (2*N* *_e_*). Critically, however, this segregational variance is not expected to differ between MA and ancestral control lines, because the ancestral control had been inbred for sufficient generations to achieve mutation-selection-drift equilibrium prior to the initiation of MA [14], [15].

The variance in the CV_E_ can be partitioned into among-line and within-line (among-gene) components. We considered the following generalized linear model, using restricted maximum likelihood (REML) as implemented in the MIXED procedure of SAS v. 9.2: *CV_E_ = Line + Gene*. The among-gene (within line) component of variance is the residual variance and was estimated for each line separately (REPEATED *Gene*/GROUP = *Line*). Degrees of freedom were determined by the Kenward-Rogers method. In principle, line is a random effect (and thus a true variance component), but given that the control “treatment” (i.e., the N2 line) is unreplicated, we treat line as a fixed effect. Our prior null hypothesis was that, averaged over all >7000 genes, the mean CV of the ancestral N2 line does not differ from the mean CV of the MA lines, i.e., the test is set up as a contrast of the N2 mean vs. the mean of the MA lines. However, the observation that the mean CV_E_ is largest in N2 suggests a different test, given the large amount of data: does the mean CV_E_ of N2 differ from the MA line with the largest mean CV_E_ (MA24)? We assessed this hypothesis using the linear model given above with only N2 and MA24 included.

The previous test addresses the hypothesis of different means, i.e., is there a directional change in the environmental variance, averaged over all genes (ΔM in the MA parlance; [16], p. 341)? A different question concerns the variation among genes in the environmental variance (CV_E_ or V_E_), in which case the null hypothesis of interest is that the among-gene component of variance does not differ between N2 and the most variable MA line. We addressed that question by likelihood-ratio test of the model with the among-gene (residual) variance estimated separately for N2 and the MA line with the greatest among-gene variance (MA99) lines vs. a model with a common among-gene variance.

The preceding analyses do not explicitly consider gene identity (analogous to an unpaired t-test), but in fact the results are identical if gene identity is explicitly considered. To show this, calculate the CV_E_ (or V_E_) for each gene in each line as above and subtract the N2 value from the mean of the four MA line values to give a gene-specific deviation. The mean gene-specific deviation is identical to the difference between the N2 and MA line means.

Denver et al. [12] identified 686 of the 7056 genes that had accumulated significant mutational (among-line) variance (V_M_). To investigate the relationship between genetic and environmental variance we considered the following general linear model: *CV_E_ = sigV_M_ + line + gene*, where *sigV_M_* is a fixed effect representing significant *V_M_* or not. Residual variance was estimated for each line separately. Unfortunately, models in which the *sigVm x line* interaction was included failed to converge, as did models in which the residual variance was estimated for each *line/sigV_M_* group separately.

Since the above analyses were conducted on normalized data, a concern is that the CV may over-correct for differences in scale. We therefore repeated the above analyses using the raw within-line variance (V_E_).

## Results and Discussion

**Figure 1:**
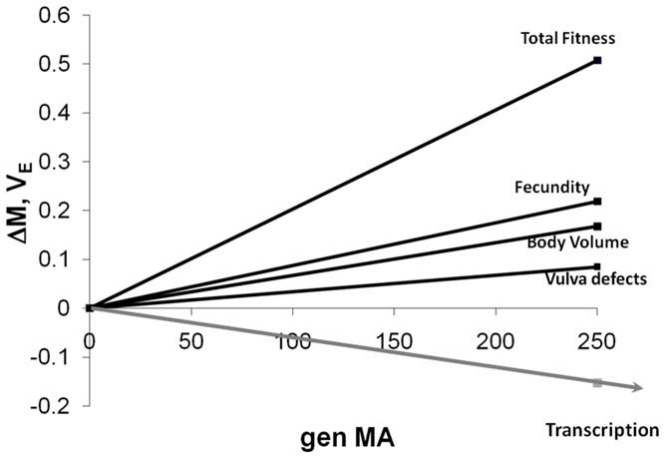
Relative rates of change (ΔM) of environmental variance. Expressed relative to the ancestral mean for Total Fitness [7], Fecundity [7], Body Volume [7], Vulval Defects [8], and Transcript Abundance (this study). V_E_ for all traits except vulval defects is expressed as the coefficient of variation. For vulval defects V_E_ represents the rate of increase in mean frequency of vulval defects and has been forced through the origin. All traits except body volume have been averaged over two sets of MA experiments using the N2 strain, including the lines used in this study; body volume is from the experiment reported in [24].

To investigate the relationship between gene expression and phenotypic robustness, we compared the environmental (residual) component of variance of transcript abundance for >7000 genes in four MA lines and their common ancestor (N2). Contrary to our prior expectation, on average the common ancestor has the most environmental variance, not the least (Table 1; Supplemental Figure S1). This result holds regardless of whether the data are scaled by the mean (CV_E_) or not (V_E_), whether the set of genes had accumulated significant V_M_ or not, and whether gene identity is considered or not. Moreover, in all cases N2 also exhibits the greatest among-gene (residual) variance. Simply put, whereas deleterious mutations unambiguously de-canalize life history (lifetime reproductive success), morphology (body volume) and vulva development, the evidence from this study clearly suggests that mutation accumulation reduces variability in gene expression (Figure 1).

**Table 1:** Measures of environmental variation in gene expression for MA and ancestral control (N2) lines.

Variable	MA24	MA41	MA83	MA99	MA mean	N2
CV_E_						
Sig V_M_	8.5796 (0.0984)	4.5358 (0.0574)	8.0409 (0.0703)	8.5659 (0.0897)	7.4306 (0.0416)	9.2155 (0.1059)
no V_M_	8.4227 (0.0270)	4.4781 (0.0186)	8.1387 (0.0220)	8.2868 (0.0335)	7.3316 (0.0141)	8.8053 (0.0358)
Total	8.4380 (0.0257)*	4.4837 (0.0177)	8.1292 (0.0210)	8.3139 (0.0315)	7.3412 (0.0133)	8.8452 (0.0339)*
V_E_						
Sig V_M_	0.9035 (0.0200)	0.3035 (0.0081)	0.7687 (0.0159)	0.9032 (0.0231)	0.7197 (0.0107)	1.1075 (0.0323)
no V_M_	0.8041 (0.0058)	0.2550 (0.0024)	0.7113 (0.0046)	0.7956 (0.0077)	0.6415 (0.0032)	0.9298 (0.0094)
Total	0.8138 (0.0056)*	0.2597 (0.0023)	0.7169 (0.0044)	0.8061 (0.0074)	0.6491 (0.0031)	0.9471 (0.0100)*
V_GENE_, CV_E_						
Sig V_M_	4.8548 (0.2623)	2.2635 (0.1223)	3.3897 (0.1832)	5.5179 (0.2982)	4.0065 (0.1082)	7.7000 (0.4161)
no V_M_	4.6283 (0.0820)	2.1985 (0.0390)	3.0734 (0.0545)	7.1597 (0.1260)	4.2650 (0.0378)	8.1535 (0.1445)
Total	4.6518 (0.0783)	2.2048 (0.0371)	3.1045 (0.0523)	7.0061 (0.1180)‡	4.2418 (0.0357)	8.1231 (0.1368)‡
V_GENE_,V_E_						
Sig V_M_	0.2755 (0.0149)	0.0455 (0.0025)	0.1773 (0.0094)	0.3662 (0.0198)	0.2161 (0.0058)	0.7150 (0.0387)
no V_M_	0.2107 (0.0037)	0.0354 (0.0096)	0.1338 (0.0024)	0.3824 (0.0068)	0.1906 (0.0017)	0.5675 (0.0101)
Total	0.2178 (0.0034)	0.0366 (0.0006)	0.1379 (0.0023)	0.3818 (0.0064)∥	0.1935 (0.0016)	0.5846 (0.0098)∥

Values in the column labeled “MA mean” are the mean of the four MA lines. Abbreviations in the column “Variable” are: CV_E_: Coefficient of Variation; sig V_M_: the set of genes for which there was significant mutational variance in [12]; no V_M_: the set of genes for which there was not significant mutational variance in [12]; Total: averaged over all genes; VE: raw (unscaled) environmental variance; V_GENE_: among-gene component of variance. See Methods for details of calculations.

* - N2<MA24, REML pseudo-F test, P<0.0001.

‡ - N2<MA99, Likelihood Ratio test, χ^2^ = 38.5, df = 1, P<0.0001.

∥ - N2<MA99, Likelihood Ratio test, χ^2^ = 317.7, df = 1, P<0.0001.

One MA line (MA41) is consistently much less variable, both in terms of mean CV_E_ and V_GENE_, than the other three MA lines. There is no obvious reason one line should be so much less variable than the others, but the presence of MA lines with extreme phenotypic values is the norm in MA experiments (e.g., [17]) and there is no obvious reason to expect that environmental variation in gene expression should be exceptional. We emphasize that the conclusion of decreased variation in MA lines relative to the ancestor does not depend on the inclusion of the MA41 line.

Interestingly, there is a significant effect of V_M_; on average, genes that accumulated significant V_M_ also showed greater environmental variance than those genes without significant V_M_. Similar result were previously reported for *Drosophila melanogaster* [18] and *Saccharomyces cerevisiae* [19]. The positive association of V_E_ with V_G_ is consistent with the idea that genetic and environmental canalization may have common underlying mechanisms [20], [21], [22]. The positive association between V_M_ and V_E_ has an important implication, because theory predicts that genetic robustness is much more likely to evolve via correlated responses to selection for environmental robustness than via direct selection [11], [20].

Although these results are internally completely consistent, there are three inherent limitations of this study that limit the robustness of the conclusions. First, and most obviously, there are only four MA lines, and it is certainly possible that for some reason gene expression in those lines differs randomly from the true MA mean. Second, the ancestral control was not subdivided into sublines ([16], p. 332), so we cannot assess the extent of residual among-line variation in the ancestor. In principle, it is expected that both the ancestral control and the MA lines are at mutation-drift equilibrium, so the residual genetic (segregational) variance is not expected to differ between MA and control lines [15]. However, among-line variance may result from non-genetic factors; e.g., see figure 3 in [23]. Finally, the environmental variance we report here includes both the among-individual variance and a fraction of the among-plate variance. This variance is certainly due to variation in some aspect of the environment, but strictly speaking, it is not what quantitative geneticists typically mean by V_E_, which in usual circumstances is the variation among individuals and is what is reported for other traits in these lines [7], [8].

Given these caveats, it seems unwarranted to speculate too much about the potential biological causes of the observed reduction in environmental variance with MA. Nevertheless, these results are potentially extremely important and should be taken seriously, in particular as motivation for further experimental work. The reduction in variability of gene expression with MA is exactly what is predicted if the underlying controls of phenotypic robustness are at least partly at the level of gene expression. If this result turns out to be general, it points toward a mechanistic explanation for an observation that has puzzled biologists for many decades: the increase in environmental variance of morphological traits with mutational and environmental stress.

## Supporting Information

Figure S1 Frequency distributions of CVE of N2 and MA lines. Each gene is a single data point. The distribution of N2 (open bars) is shown plotted against (A) MA24 (B) MA41 (C) MA83 (D) MA99 (E) the mean of the four MA lines.
(4.04 MB PDF)

Table S1 This file contains the normalized data analyzed in this study (datasheet “Data”) and a table legend (datasheet “README”).
(5.58 MB XLS)
