# Supplementary material for: Spontaneous Mutations Decrease Sensitivity of Gene Expression to Random Environmental Variation in Caenorhabditis elegans
Source: PLoS One. 2010 Jan 18;5(1):e8750. doi: 10.1371/journal.pone.0008750 (PMC2807463; doi:10.1371/journal.pone.0008750)
Supplement: Figure S1 — Frequency distributions of CVE of N2 and MA lines. Each gene is a single data point. The distribution of N2 (open bars) is shown plotted against (A) MA24 (B) MA41 (C) MA83 (D) MA99 (E) the mean of the four MA lines. (4.04 MB PDF) [file pone.0008750.s001.pdf]

Figure S1 - Frequency distributions of CV_E of N2 and MA lines. Each gene is a single data point. The distribution of N2 (open bars) is shown plotted against (A) MA24 (B) MA41 (C) MA83 (D) MA99 (E) the mean of the four MA lines.

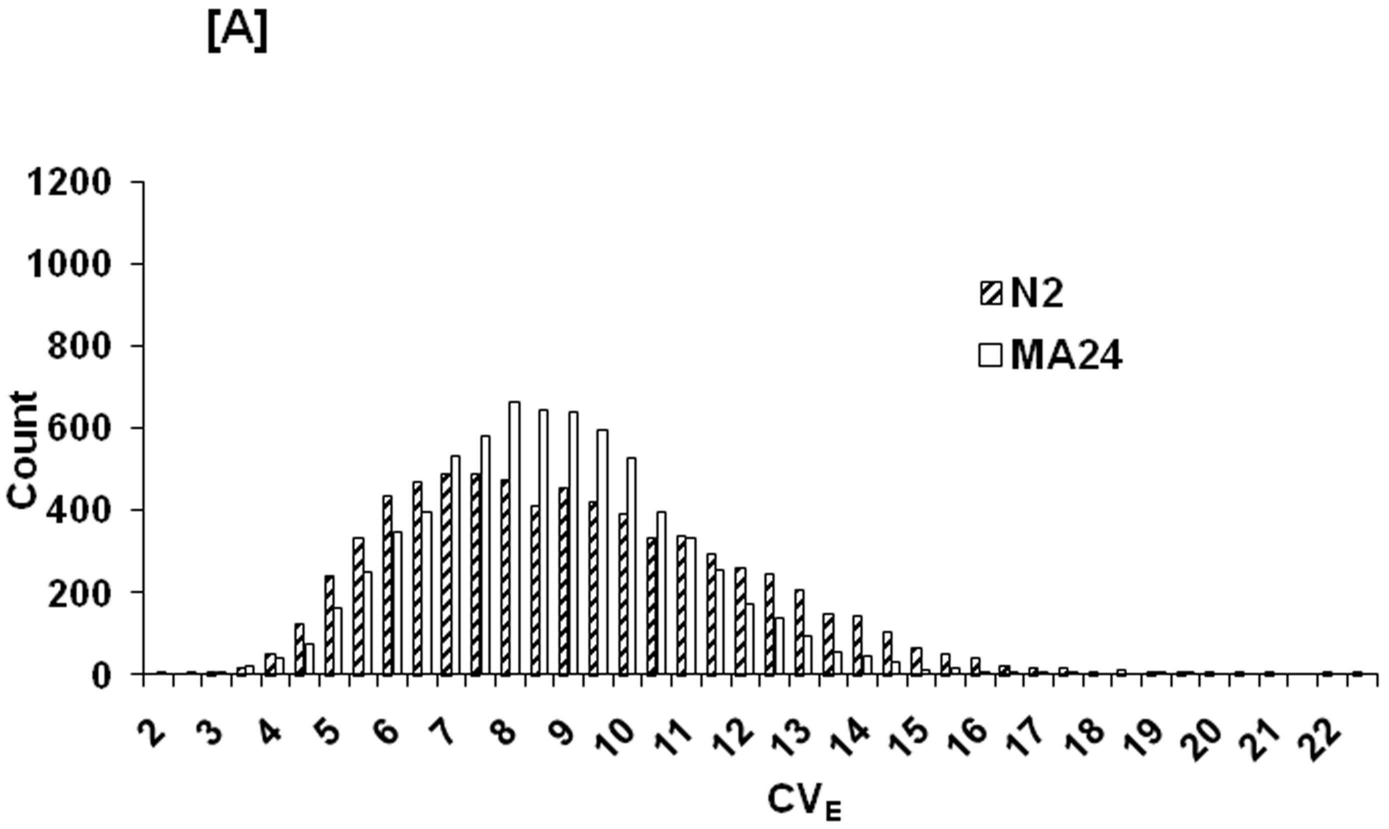
[A]
1200
1000
800
600
400
200
0
Count
N2
MA24
2
3
4
5
6
7
8
9
10
11
12
13
14
15
16
17
18
19
20
21
22
CV_E

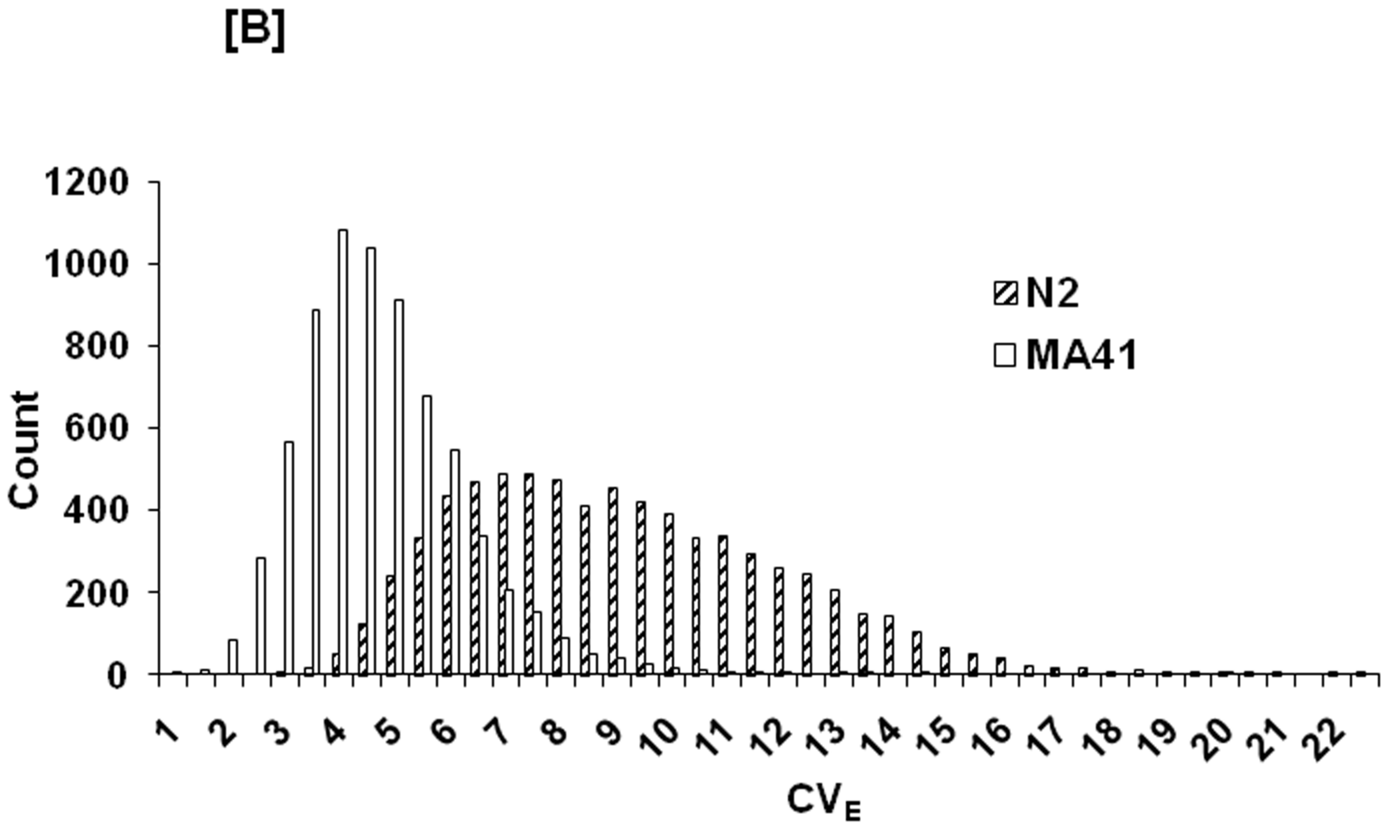

[B]
N2
MA41
Count
1200
1000
800
600
400
200
0
1
2
3
4
5
6
7
8
9
10
11
12
13
14
15
16
17
18
19
20
21
22
CV_E

[C]

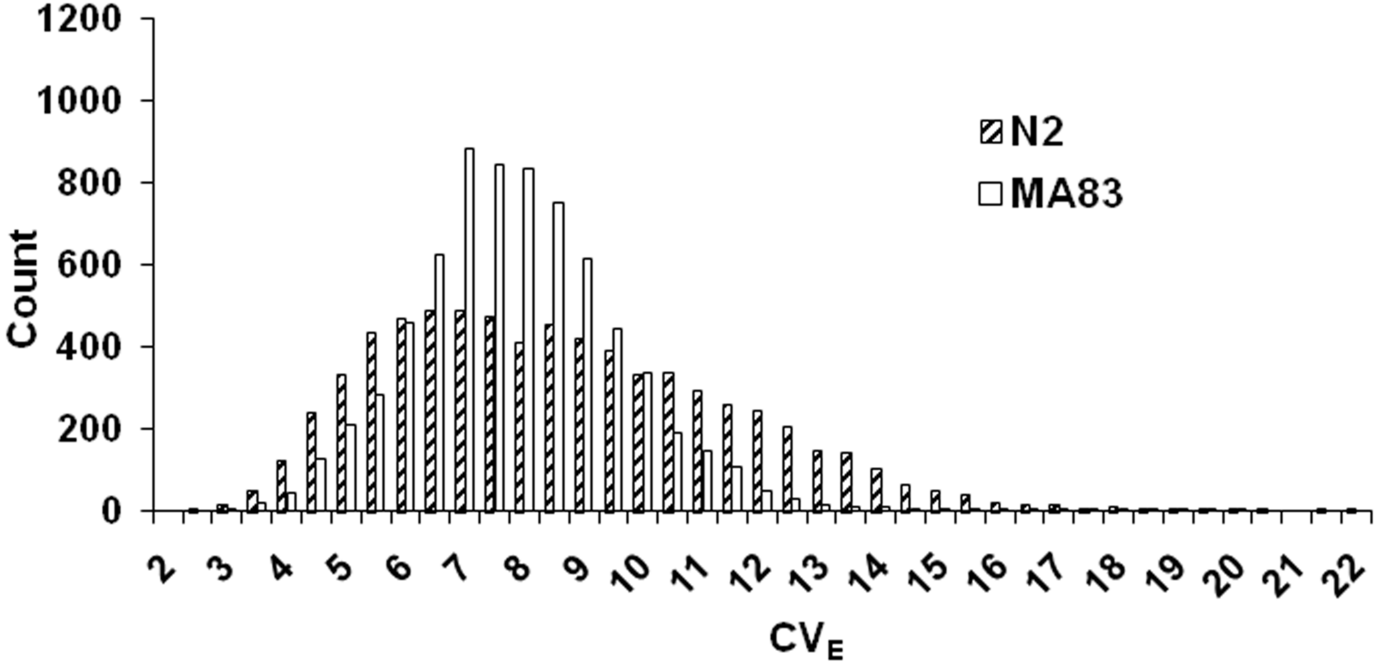

1200
1000
800
600
400
200
0
Count
N2
MA83
2
3
4
5
6
7
8
9
10
11
12
13
14
15
16
17
18
19
20
21
22
CV_E

[D]

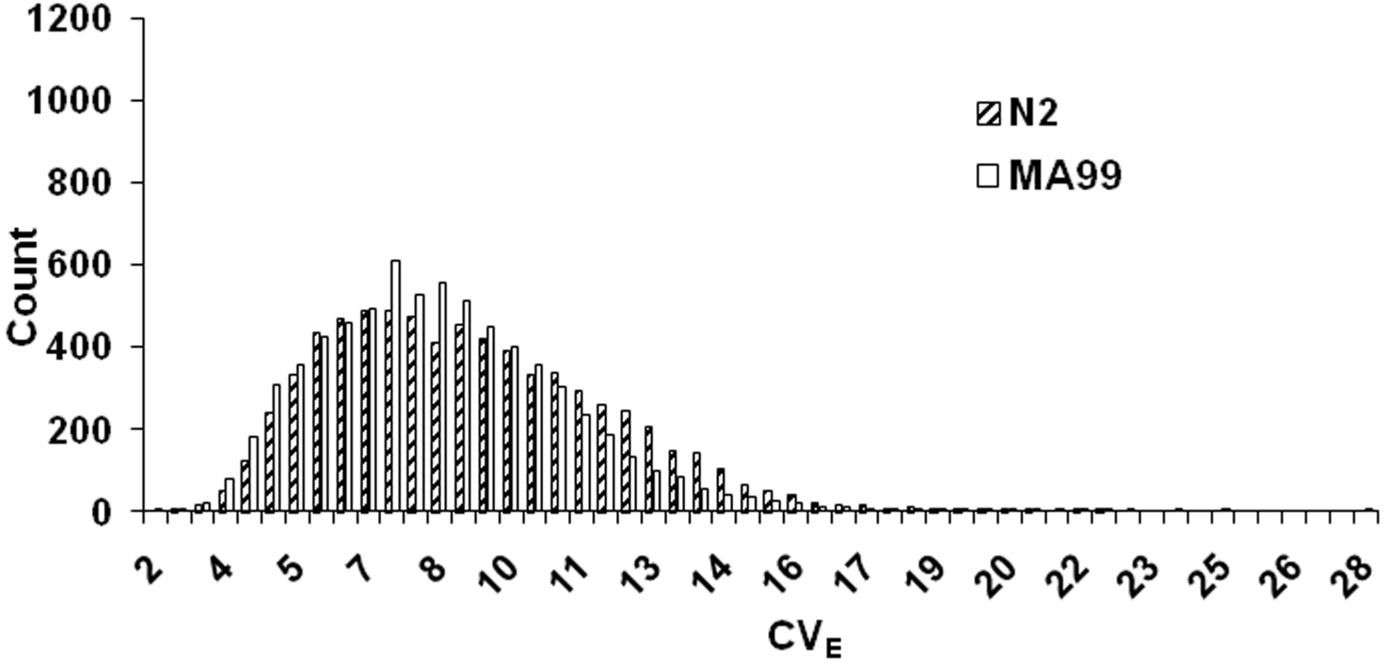
1200
1000
800
600
400
200
0
Count
N2
MA99
2
4
5
7
8
10
11
13
14
16
17
19
20
22
23
25
26
28
CV_E

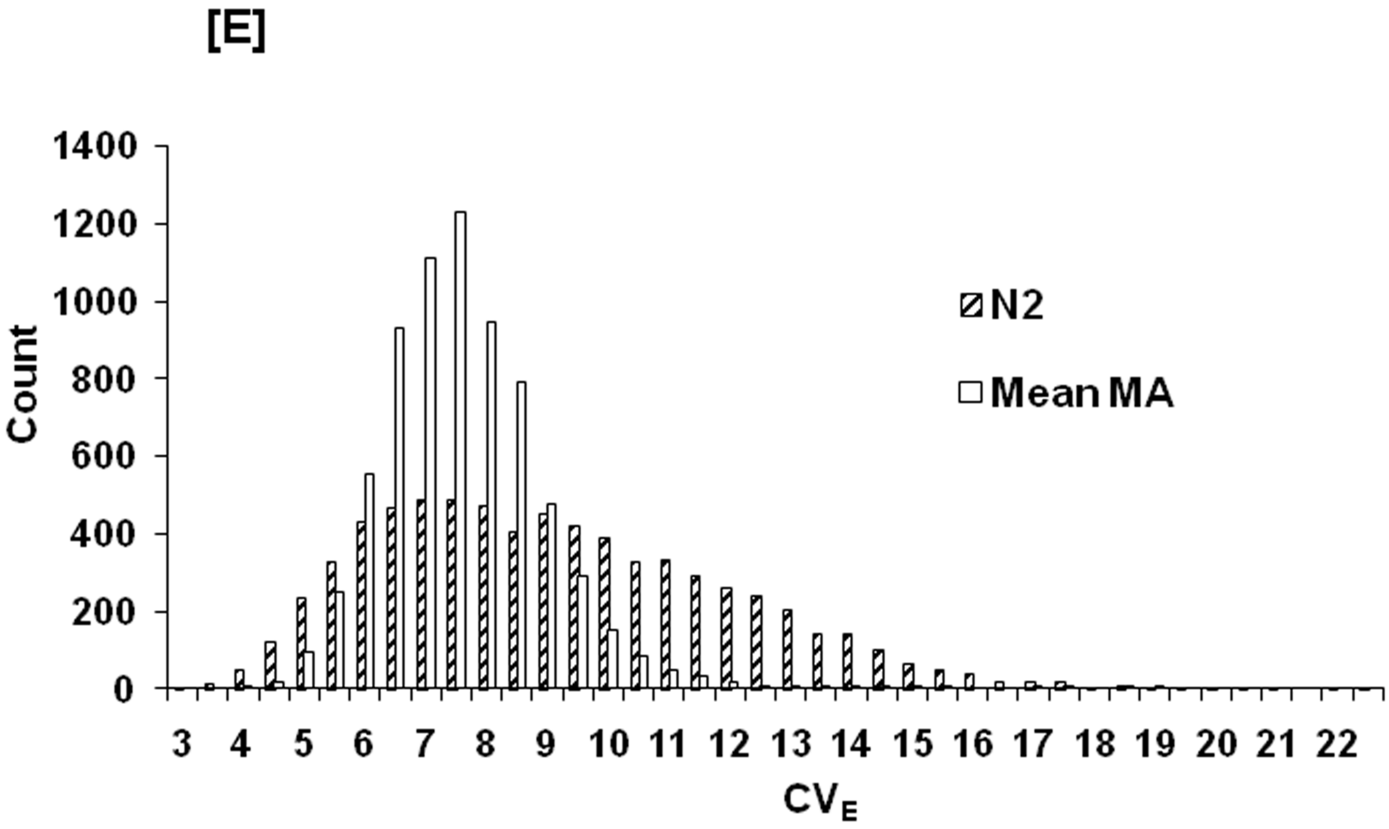
[E]
Count
1400
1200
1000
800
600
400
200
0
N2
Mean MA
3
4
5
6
7
8
9
10
11
12
13
14
15
16
17
18
19
20
21
22
CV_E
